# Intensive Family-Centered Rehabilitation and Motor Outcomes in a Child with Global Developmental Delay: A Case Report

**DOI:** 10.3390/children13091218

**Published:** 2026-09-09

**Authors:** Jelena Erceg, Svetislav Polovina, Andrea Polovina, Ema Dobrijević, Romana Gjergja Juraški

**Affiliations:** 1 Polyclinic “Prof. dr. sc. Milena Stojčević Polovina”, Svetice 36, 10000 Zagreb, Croatia; jelena.erceg@poliklinika.org (J.E.); info@poliklinika.org (S.P.); ema.dobrijevic@poliklinika.org (E.D.); rgjergja@bolnica-srebrnjak.hr (R.G.J.); 2 Neuropaediatric Department, Srebrnjak Children’s Hospital, Srebrnjak 100, 10000 Zagreb, Croatia; 3 Faculty of Medicine, Josip Juraj Strossmayer University of Osijek, J. Huttlera 4, 31000 Osijek, Croatia; 4 Faculty of Health Studies, University of Rijeka, Ulica Viktora cara Emina 5, 51000 Rijeka, Croatia

**Keywords:** global developmental delay, early intervention, family-centered rehabilitation, Early Intensive Stojčević-Polovina Rehabilitation Method, Gross Motor Function Measure, cerebral palsy, Vojta method, case report

## Abstract

**Highlights:**

**What are the main findings?**

• In this single case, the Early Intensive Stojčević-Polovina Rehabilitation Method (EIR-SPM) was accompanied by sustained gross motor gains in a girl with global developmental delay (GDD).
• Progress appeared more favorable than typically reported in GDD of undetermined etiology, though a single-case design cannot separate this from maturation.

**What are the implications of the main findings?**

• A family-centered structure may allow high-intensity rehabilitation to be sustained at home.
• Rehabilitation can begin before the diagnostic work-up is complete.

**Abstract:**

**Background**: Global developmental delay (GDD) affects multiple domains of early childhood development, including gross motor, cognitive and communication skills. Early, intensive, family-centered rehabilitation is considered key to optimizing functional outcomes in affected children. **Case Presentation**: We report a female child with GDD who began rehabilitation at our institution at 15 months of age, presenting with generalized hypotonia with superimposed fluctuating episodes of hypertonia, poor postural control, absent independent sitting, markedly reduced spontaneous motor activity, and associated cognitive and communication delay. Brain MRI at 7 months showed no parenchymal abnormality, with mildly enlarged extracerebral cerebrospinal fluid spaces and ventricular system. The metabolic and genetic evaluation performed so far, including microarray/MLPA-based screening for common microdeletion syndromes and SMN1/SMN2 genotyping, has not identified a specific underlying etiology. Diagnostic work-up is ongoing. Rehabilitation was delivered as a comprehensive, multidomain program; this report focuses specifically on the child’s motor progression. **Intervention**: The child underwent the Early Intensive Stojčević-Polovina Rehabilitation Method (EIR-SPM), a high-intensity, continuous approach for children with cerebral palsy, at-risk infants, and other developmental disabilities, built on parental education enabling home-based continuity of therapy. Rehabilitation focus is selected according to the child’s optimal developmental stage—the milestone showing the least abnormal movement patterns and muscle tone—rather than chronological age, with positions progressively adjusted following the trajectory of typical motor development described by Vojta. **Results**: Gross motor function, monitored using the Gross Motor Function Measure–88 (GMFM-88) at four assessment points from 15 months to 6 years 6 months of age, improved progressively from 10.8% to 48.9%, 64.7%, and finally 73.9%. The child achieved independent kneeling, reciprocal crawling, independent sitting in all positions, independent standing and assisted stepping. **Conclusions**: In this child with GDD of undetermined etiology, more than five years of intensive, family-centered rehabilitation according to the EIR-SPM were accompanied by substantial and sustained gains in gross motor function and functional independence. This report suggests that meaningful progress remains achievable even when rehabilitation begins later than the period considered optimal within the EIR-SPM framework, and that a family-centered structure may be what makes therapy of this intensity and duration sustainable.

## 1. Introduction

Global developmental delay (GDD) is defined as a significant delay in two or more of the following developmental domains: gross and fine motor function, speech and language, cognition, social and personal skills, and activities of daily living. Significant delay is defined as performance of two or more standard deviations below the mean on age-appropriate, standardized, norm-referenced testing [1]. The reported prevalence of GDD is around 1% to 3% [2]. Establishing an underlying etiology is often difficult, and many children remain without a definitive diagnosis [3]. Genetic causes account for roughly a quarter to a half of identified cases [4], and children with poorer motor profiles in particular may warrant prioritized genetic testing [5]. When the etiology is established, prognostication and family counseling are better supported than when no specific cause is found [6]. Early identification and intervention can improve a child’s prognosis and reduce the likelihood of later intellectual disability [7]. The evidence base for motor rehabilitation is most developed in cerebral palsy, where a broad range of interventions exists: constraint-induced movement therapy, environmental enrichment, fitness training, goal-directed training, hippotherapy, home programs, mobility training, strength training, task-specific training, treadmill training, partial body-weight-support treadmill training, and others [8]. In GDD specifically, far fewer intervention studies exist. Where evidence is available, service intensity, early timing, and family involvement have been identified as key determinants of developmental outcome [9], and a parent-implemented program produced greater developmental gains than standard care in a multicenter controlled study of infants with GDD (locomotor, personal–social, and language outcomes) [7].

The Early Intensive Stojčević-Polovina Rehabilitation Method (EIR-SPM) is a rehabilitation approach designed for children with cerebral palsy (CP), children at risk of developing CP, and children with other developmental disabilities [10]. The EIR-SPM begins as soon as abnormal development is recognized and is delivered by trained caregivers at home, allowing an intensity and duration that institutional therapy cannot sustain. In this report, high intensity refers to the daily dose of therapy sustained over years—approximately three hours of EIR-SPM and three 15 min Vojta sessions per day—rather than to a single session’s effort or difficulty. This dose was monitored through the parents’ daily records and regular clinical review.

Contemporary frameworks emphasize active, child-initiated, task-oriented practice and participation-focused goals [8,11]. The EIR-SPM shares several of these principles—it is delivered through the child’s spontaneous activity and play, is developmentally staged, and is intensive and family-delivered, while differing in its explicit use of a milestone-based developmental sequence rather than task-specific training toward discrete functional goals.

Long-term, systematic quantification of motor outcome in children with GDD of undetermined etiology is not commonly reported. Here we present the case of a child with GDD of undetermined etiology who was enrolled in the EIR-SPM program. Although the intervention addressed all developmental domains, this report concentrates on the child’s motor progress and on the changes in rehabilitation strategies and tasks over more than five years of rehabilitation.

The Gross Motor Function Measure (GMFM) is a standardized, criterion-referenced instrument originally developed and validated to quantify gross motor function and its change over time in children with cerebral palsy; it has since been applied more broadly in children with motor impairment of other etiologies [12,13,14]. Its responsiveness to change makes it particularly suitable for documenting motor progress across an extended rehabilitation period. Given that cerebral palsy remained within the differential diagnosis in our patient, the GMFM was selected to systematically monitor gross motor function throughout rehabilitation, allowing the child’s motor trajectory to be quantified and examined in detail. Nevertheless, validation of the GMFM in conditions other than CP remains limited despite its widespread use, and results should be interpreted cautiously until more robust validation is established [13]. Gross motor function corresponds to the activity level of the International Classification of Functioning, Disability and Health (ICF), which provides a useful framework for situating rehabilitation targets and outcomes across body functions, activities, and participation.

## 2. Case Presentation

The patient is a female child who began rehabilitation at our institution at 15 months of age because of GDD. On initial clinical examination, she presented with generalized hypotonia with superimposed fluctuating episodes of hypertonia, poor postural control, and markedly reduced spontaneous motor activity. The fluctuating episodes of hypertonia were clinically assessed and did not have the features of dystonia. At 15 months, her gross motor function was at an early level: she was able to roll to prone but with insufficient dissociation of the lower extremities, and in prone she showed deficient righting reactions, at times pushing up onto stiffly extended arms. She was not able to sit independently and could not maintain a sitting position. On clinical assessment, delays relative to age were also evident in the cognitive and communication domains, consistent with GDD. Although the formal definition of GDD requires performance ≥2 SD below the mean on standardized testing [1], reliable psychometric assessment is frequently not feasible in very young children, and the diagnosis in this age group rests on the summation of clinical findings across developmental domains [15].

The child lives with both parents and sisters, within a supportive extended family; both grandparents were closely involved and took part in the home rehabilitation program.

The child had received regular rehabilitation in her home country before referral to our institution.

Brain magnetic resonance imaging, performed at 7 months of age prior to referral, showed no parenchymal abnormality; mildly enlarged extracerebral cerebrospinal fluid spaces and a mildly enlarged ventricular system. Genetic and metabolic evaluation performed to date—including chromosomal microarray and MLPA-based screening for common microdeletion syndromes and SMN1/SMN2 genotyping for spinal muscular atrophy—has not identified a specific underlying etiology. Whole-exome sequencing is planned as the next diagnostic step but has not yet been completed; the diagnostic work-up therefore remains ongoing.

## 3. Intervention and Assessment

### 3.1. Intervention

The child underwent the EIR-SPM, an intensive and continuously applied rehabilitation approach for children with CP, children at risk of developing CP, and children with other developmental disabilities [10,16]. The EIR-SPM is primarily a family-centered method, in which parents or caregivers deliver the rehabilitation at home according to the training they receive. Rehabilitation began immediately after the initial assessment at our institution, where the parents received structured instruction and practical training over a period of seven days. In parallel with the EIR-SPM, the child was also enrolled in Vojta therapy [17]. These represented two complementary levels of intervention: the EIR-SPM served as the overall developmental framework, which itself follows the sequence of typical motor development described by Vojta, while Vojta reflex locomotion exercises were additionally applied as a distinct, structured technique.

Thereafter, rehabilitation was carried out daily at home according to the EIR-SPM recommendations, with individual adjustments to the child’s rhythm and capacities. During the first two years, each day comprised three rehabilitation blocks. Each block began with a 15 min session of Vojta reflex locomotion administered by the parents, followed by approximately one hour of EIR-SPM exercises, giving about three hours of EIR-SPM and three 15 min Vojta sessions per day. Vojta therapy was later discontinued after approximately two years, as discussed below. Over the same period, follow-up sessions were conducted at our institution over two days each month (except when the child was unwell) in order to monitor clinical changes, guide the parents, and adapt the program as needed. Thereafter, follow-up visits continued every three months following the same format. In addition, online consultations were available between scheduled visits whenever questions or difficulties arose. These covered the whole rehabilitation program—the EIR-SPM, the Vojta exercises, and the non-motor domains—allowing any component to be reviewed and adjusted without delay. The program was delivered by the rehabilitation team, most often by one primary therapist, which ensured continuity of clinical reasoning while allowing input from the wider team and from the specialists involved in the non-motor domains. Both parents were actively involved in the instruction and home execution of the program, with the mother taking the larger share. During the follow-up period, a younger sibling was born when the child was 4 years old; around the time of the birth, the grandmother temporarily took over the main share of the home rehabilitation.

The program was comprehensive and addressed multiple developmental domains: gross motor function, fine motor skills, speech, communication, and cognitive stimulation. Motor rehabilitation was delivered strictly according to the EIR-SPM, while rehabilitation of the other developmental domains—primarily cognitive, speech, and communication—was carried out in collaboration with specialists from the child’s country of origin, from which she traveled for rehabilitation at our institution.

The EIR-SPM intervention was based on identifying the child’s optimal developmental stage, defined as the developmental milestone or milestones at which the girl exhibited the least abnormal motor pattern and the fewest abnormalities of muscle tone. This stage did not necessarily correspond to the child’s chronological age. Parents were guided to help the child move away from the most markedly atypical movement components—for example, by correcting the quality of rolling and creeping and by encouraging a wider repertoire of postures and transitions—while promoting the child’s own spontaneous, self-initiated activity within the developmental milestone or milestones representing the current therapeutic focus. Here, greater variability meant the ability to achieve a given milestone in many different ways—for example, coming to sit through more than one movement sequence—each with as few abnormal components as possible, rather than a lower-quality or more stereotyped performance. The key aim was to maintain the child in the optimal developmental position until a normal or near-normal motor pattern became part of her spontaneous activity and mobility; the focus of therapy was then advanced stepwise to the next developmental milestone, in keeping with the sequence of typical motor development on which the EIR-SPM is based. The optimal developmental stage was identified through structured clinical observation of movement quality and muscle tone across milestones, selecting the level at which the child showed the least atypical movement and the greatest capacity for active, guided practice. This reflects a broader principle of motor learning, in which repeated, actively performed movement with sensory feedback and appropriate guidance shapes the emergence of more adaptive patterns; within the EIR-SPM this principle is framed through the neuronal group selection theory as applied in our earlier work [16].

The intervention included the following elements: application of variable pressure, primarily over the trunk, to promote postural stabilization, weight shifting with corresponding equilibrium reactions and control, facilitation of individual developmental milestones through stimulation of righting, equilibrium, and protective reactions and passive, active, and assisted-active large movements of the extremities to enhance limb mobility, as well as small passive, assisted-active, and active movements aimed at achieving selectivity of movement. All passive, assisted-active, and active movements were performed with variation in speed, force, and amplitude. Throughout the therapy, particular attention was directed toward providing appropriate sensory input to improve visual, auditory, and cognitive functioning. The entire rehabilitation procedure was carried out at home, so that therapy was effectively delivered within the child’s spontaneous activity and play.

Vojta therapy, or reflex locomotion, is a rehabilitation method in which defined pressure is applied to specific stimulation zones on the body while the child is positioned in prone, supine, or side-lying. This elicits involuntary, reproducible global motor responses—reflex creeping and reflex rolling—that activate coordinated patterns of postural control, trunk stabilization, and limb movement. Through repeated activation, these innate motor patterns are intended to become more accessible to the child’s spontaneous movement, supporting the development of righting, supporting, and equilibrium reactions [17]. 

### 3.2. Assessment

Gross motor functions were assessed using the Gross Motor Function Measure–88 (GMFM-88), a standardized, criterion-referenced observational instrument developed to quantify gross motor function and its change over time in children [12]. The GMFM-8 comprises 88 items grouped into five dimensions: (A) lying and rolling; (B) sitting; (C) crawling and kneeling; (D) standing; and (E) walking, running, and jumping. Each item is scored on a four-point ordinal scale, and a total percentage score is derived, with higher scores indicating better gross motor function [18]. All 88 items were administered at each assessment, and total and dimension percentage scores were calculated. The GMFM-88 total percentage score was used as the primary outcome. We acknowledge that the GMFM-88 percentage score is an ordinal-based summary and psychometrically less robust than the Gross Motor Function Measure–66 (GMFM-66) interval scale; however, because the GMFM-66 calibration is specific to cerebral palsy—a diagnosis not established in this child—we considered the GMFM-88 the more appropriate primary outcome. The same administered items were additionally processed with the GMFM App+ (Gross Motor Ability Estimator GMAE-3), which derives GMFM-66 interval scores through Rasch analysis [18]; these interval scores, together with their associated measurement error, are reported alongside the GMFM-88 as a complementary, supportive measure. All GMFM-88 assessments were administered by the same experienced examiner—a physical therapist trained and experienced in GMFM administration and a member of the rehabilitation team—throughout the follow-up period, ensuring consistency of scoring across the four time points.

## 4. Results

Gross motor function was monitored using the Gross Motor Function Measure (GMFM-88) at four assessment points, from initial evaluation at our institution at 15 months of age to final assessment at 6 years 6 months of age. GMFM-88 scores improved progressively from 10.8% at baseline to 48.9%, 64.7%, and finally 73.9%, paralleled by continued gains in functional mobility level. Over this period, the child achieved independent kneeling, reciprocal crawling, independent sitting in all positions, independent standing with adequate postural alignment, and assisted stepping.

**Figure 1:**
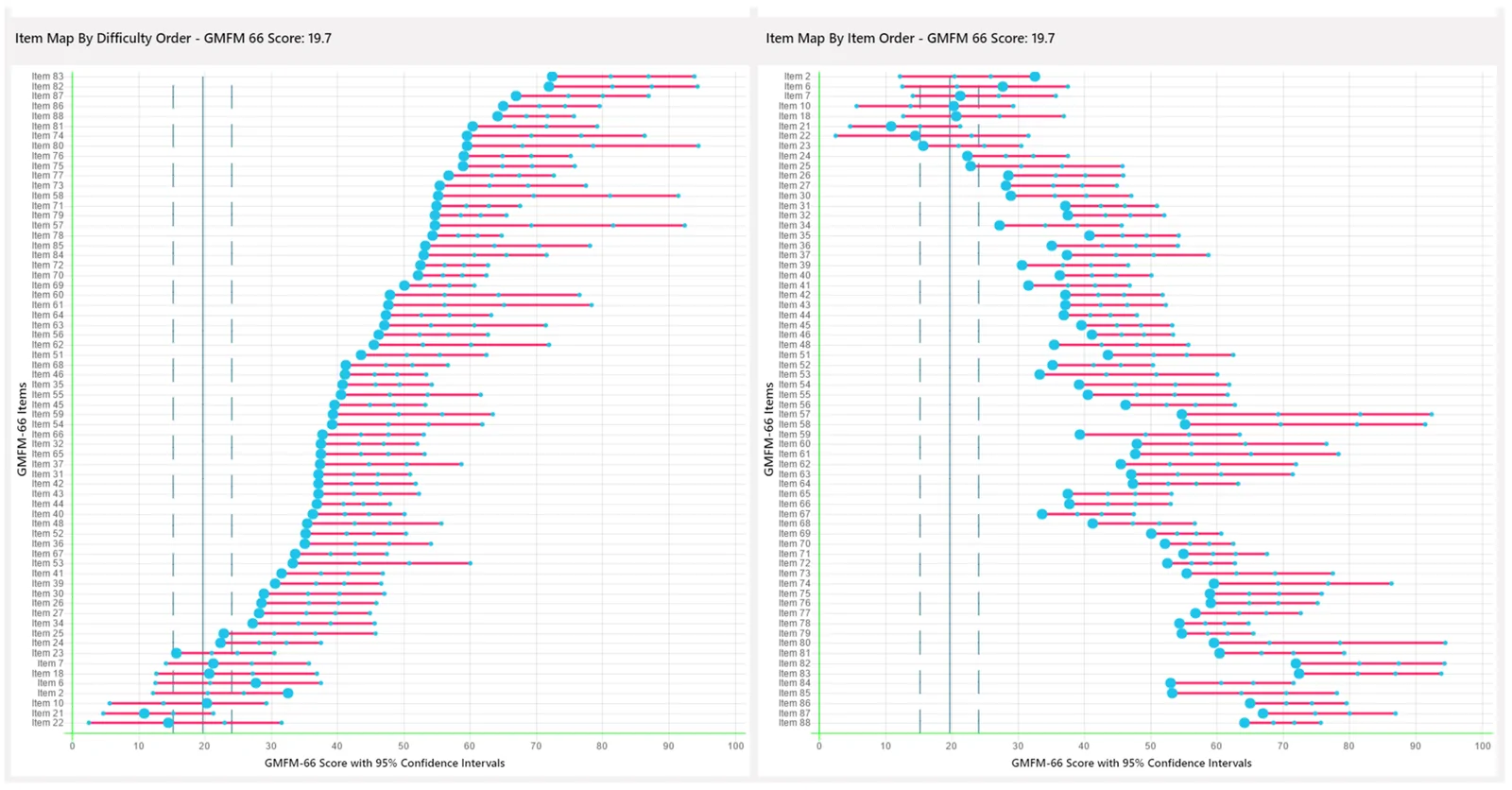
GMFM-66 of the first test: by difficulty order and by item order.

**Figure 2:**
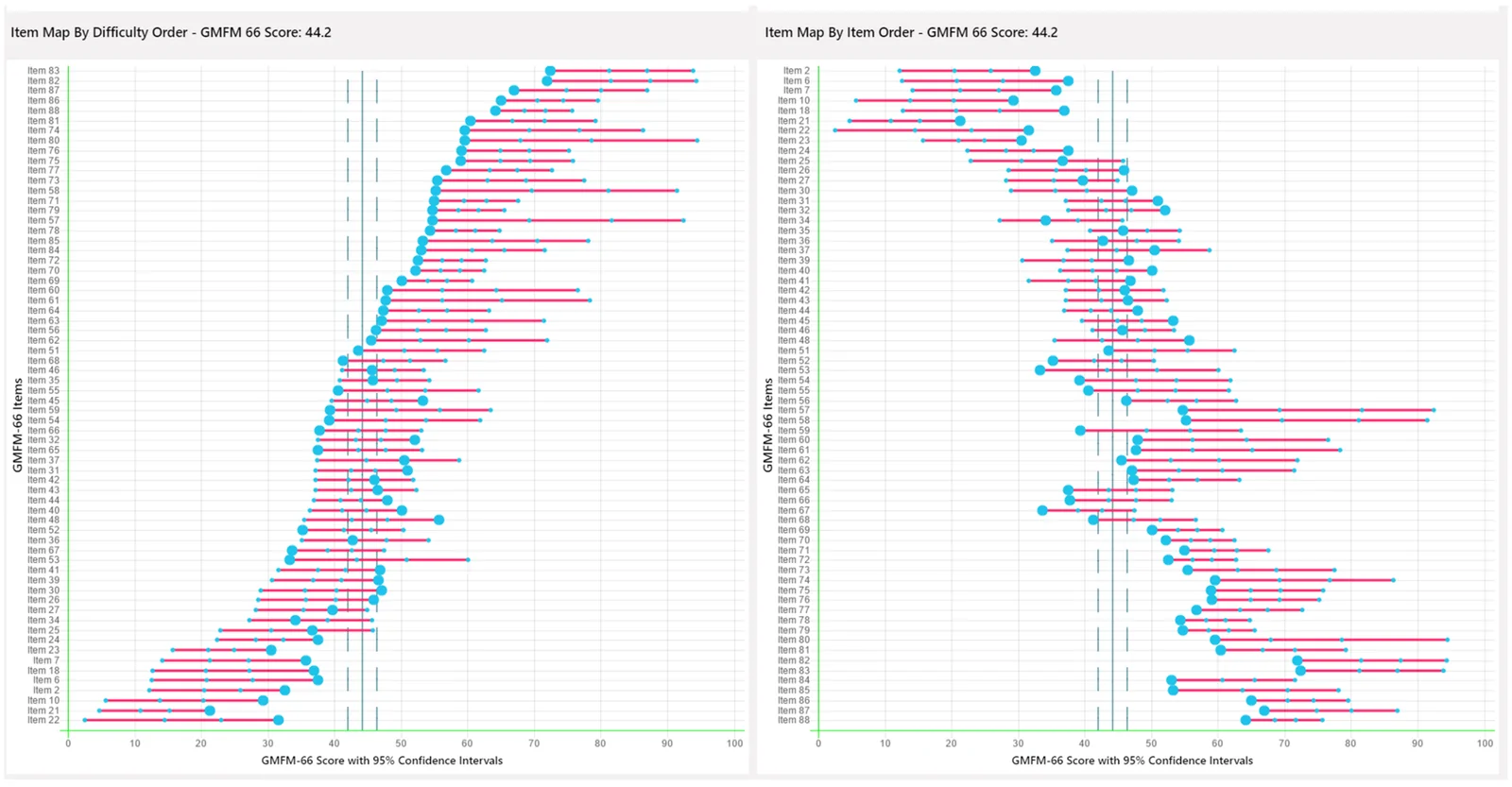
GMFM-66 of the second test: by difficulty order and by item order.

**Figure 3:**
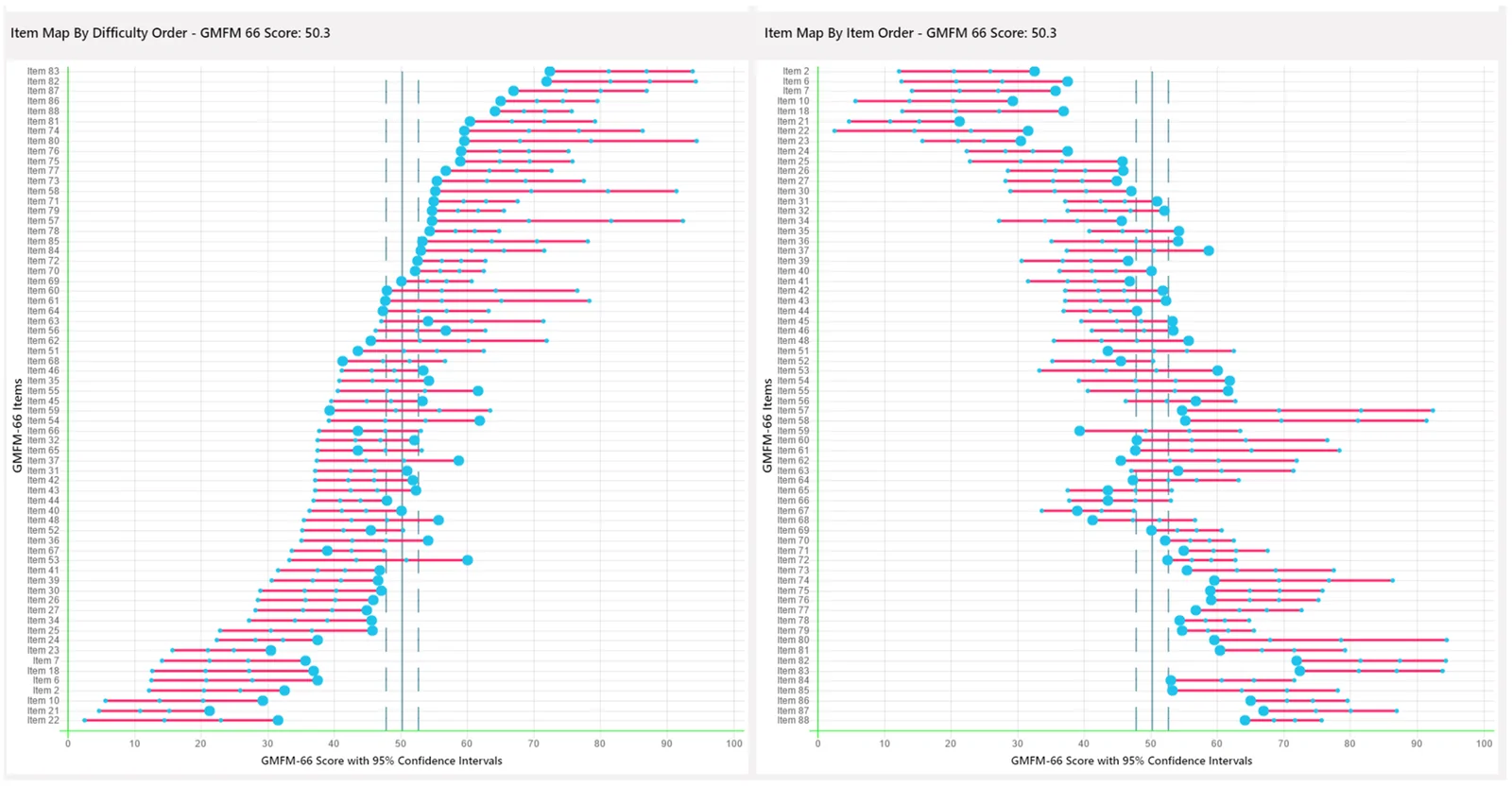
GMFM-66 of the third test: by difficulty order and by item order.

**Figure 4:**
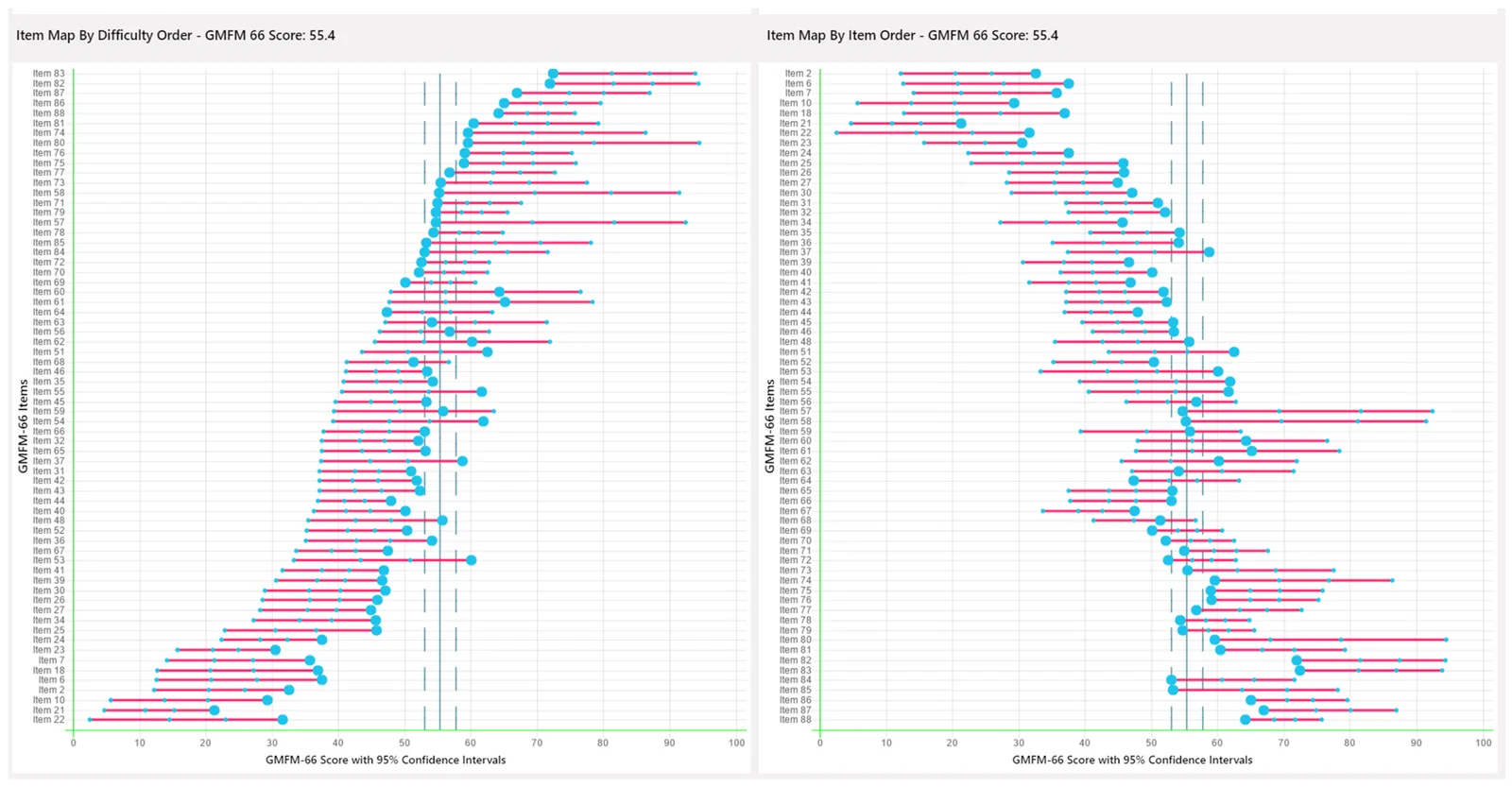
GMFM-66 of the fourth test: by difficulty order and by item order.

**Figure 5:**
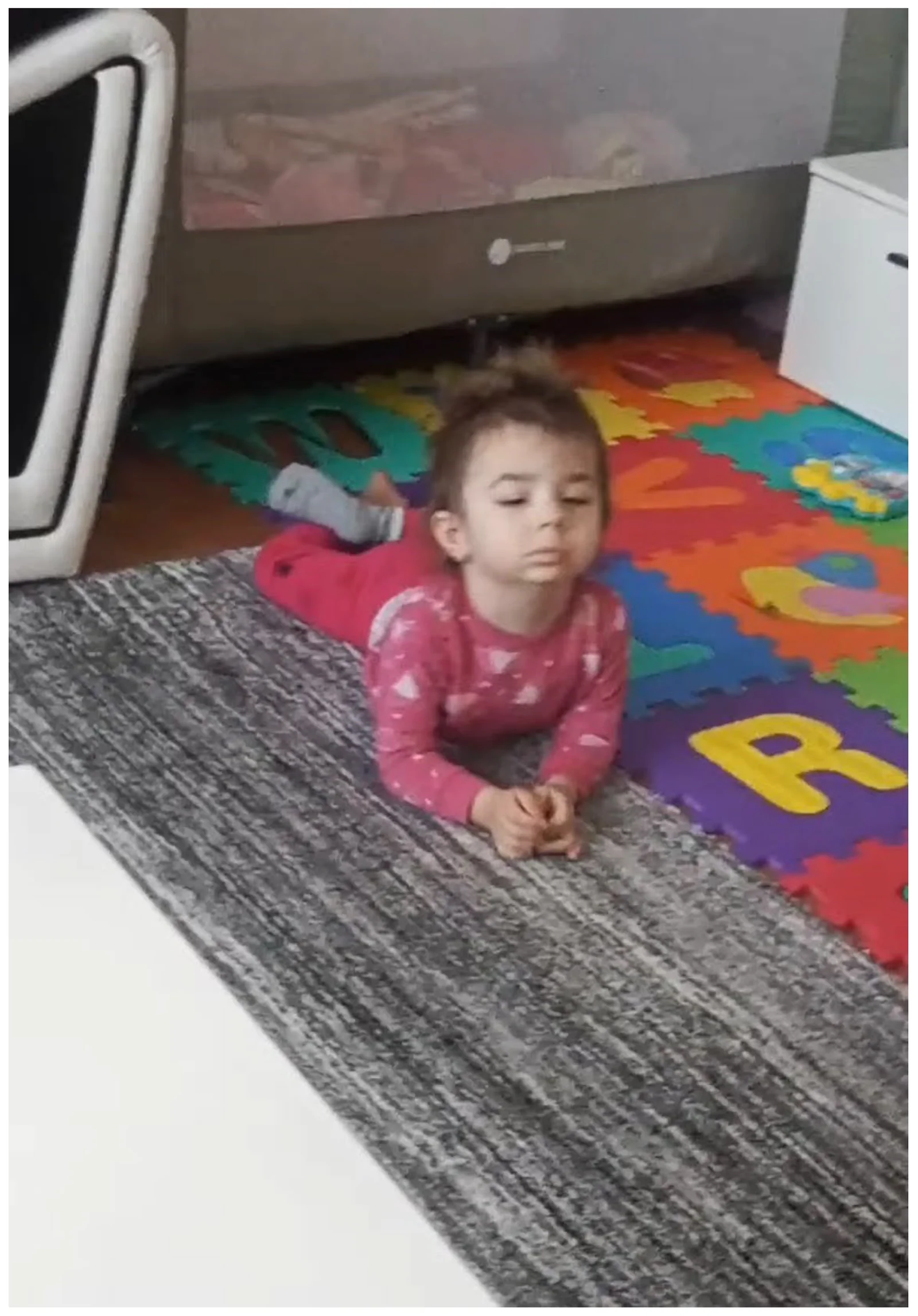
Ability to support herself on elbows in prone position.

**Figure 6:**
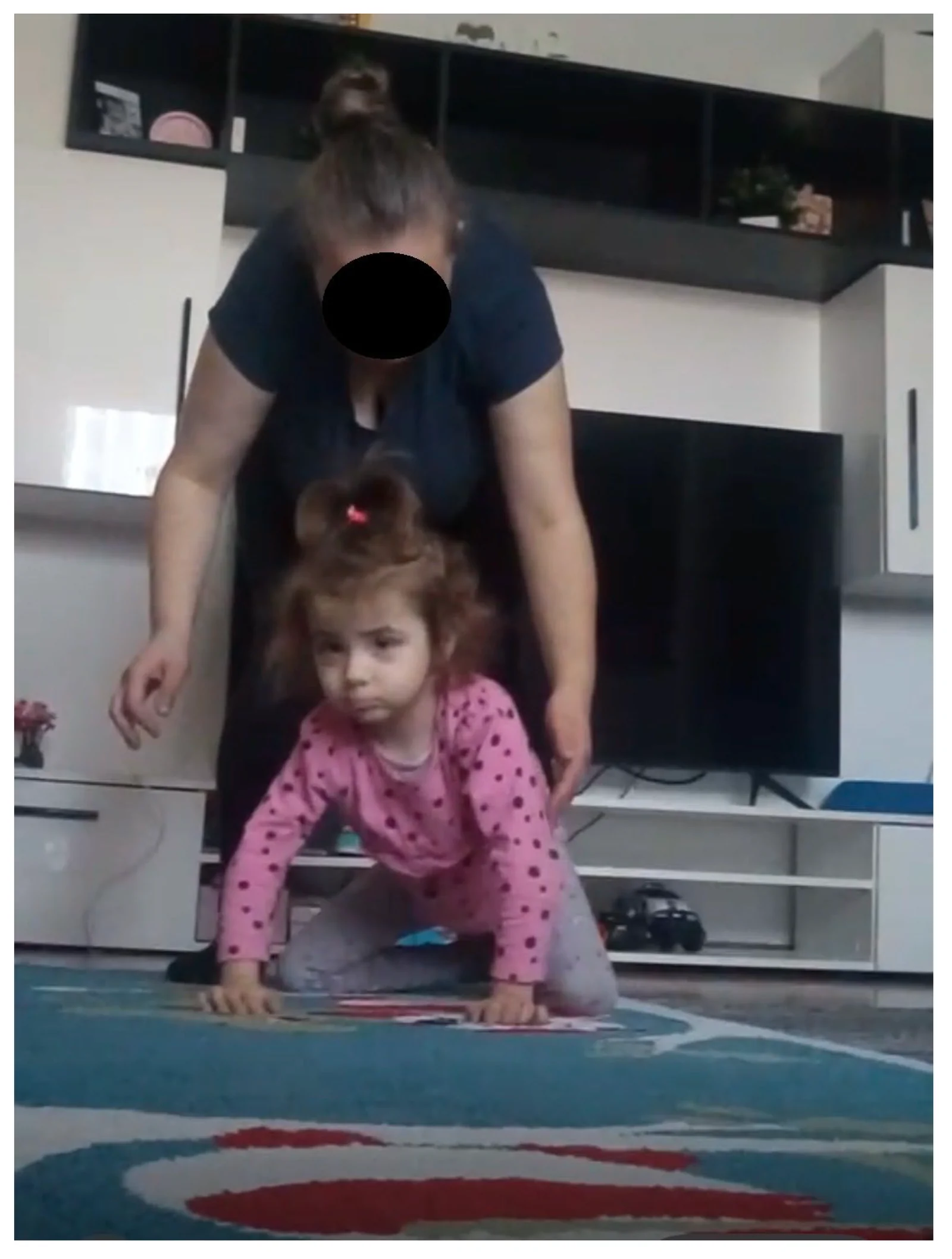
Ability for hand and knees support.

**Figure 7:**
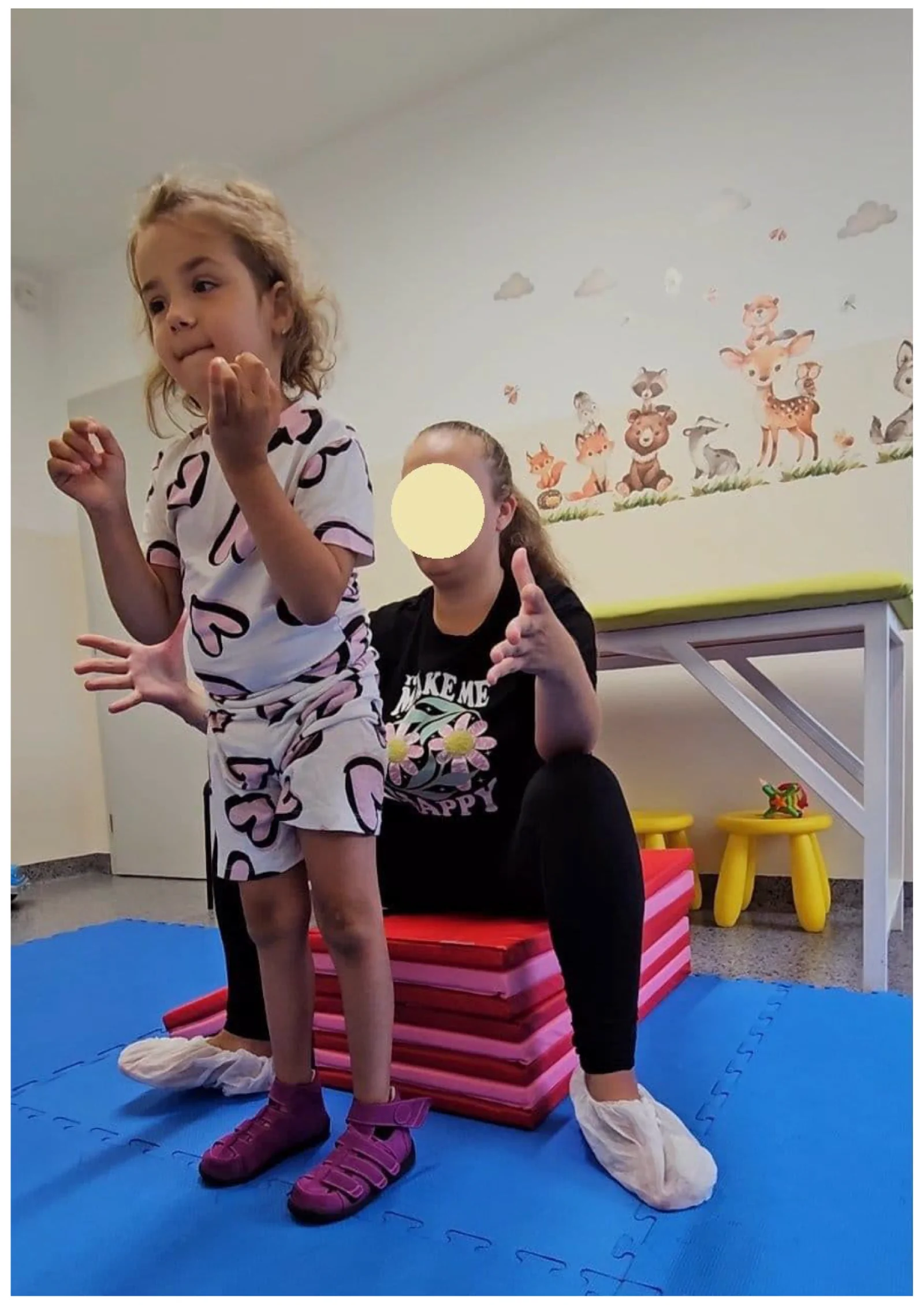
Ability to stand by herself for more than 3 s.

**Figure 8:**
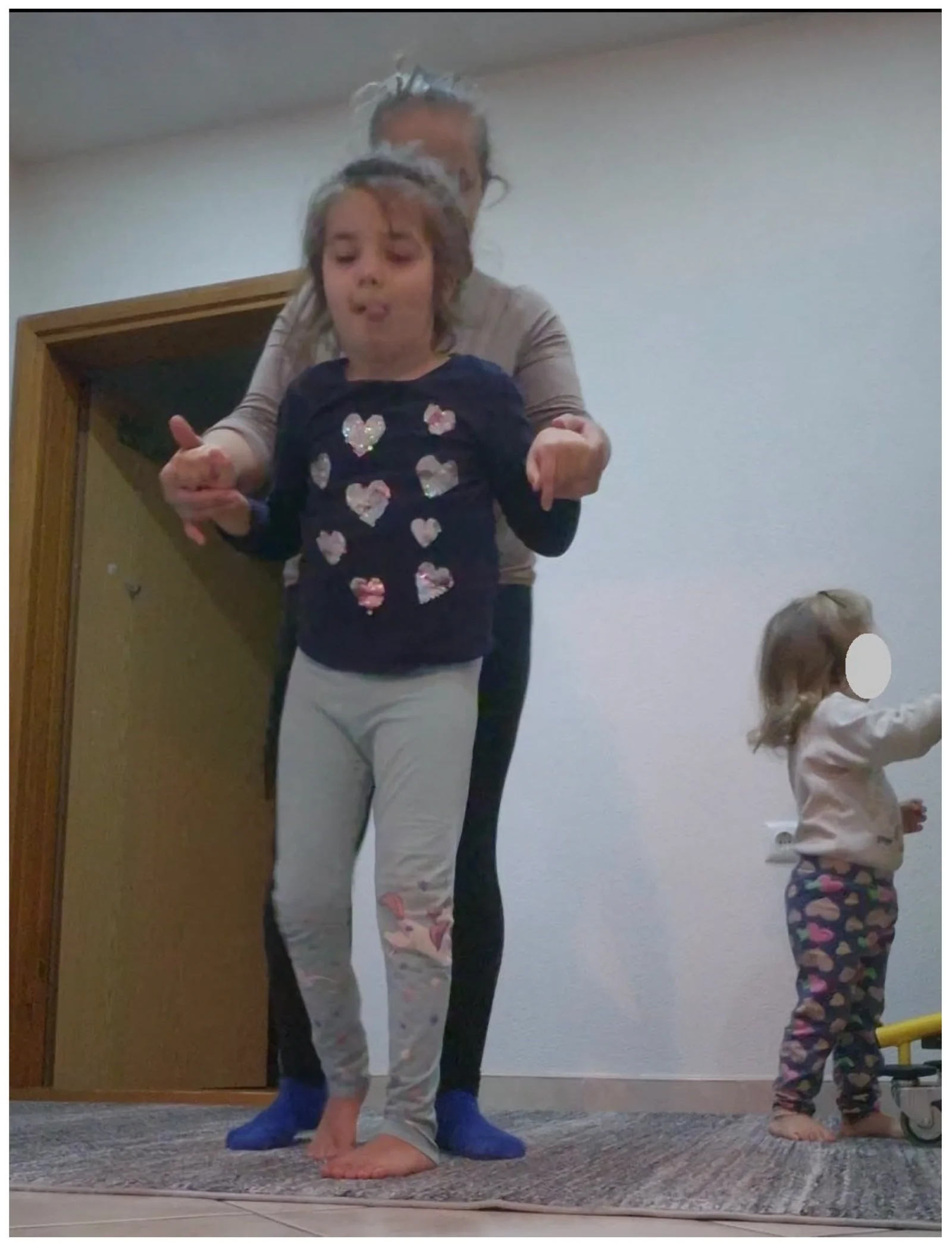
Ability to walk supported with two hands.

**Figure 9:**
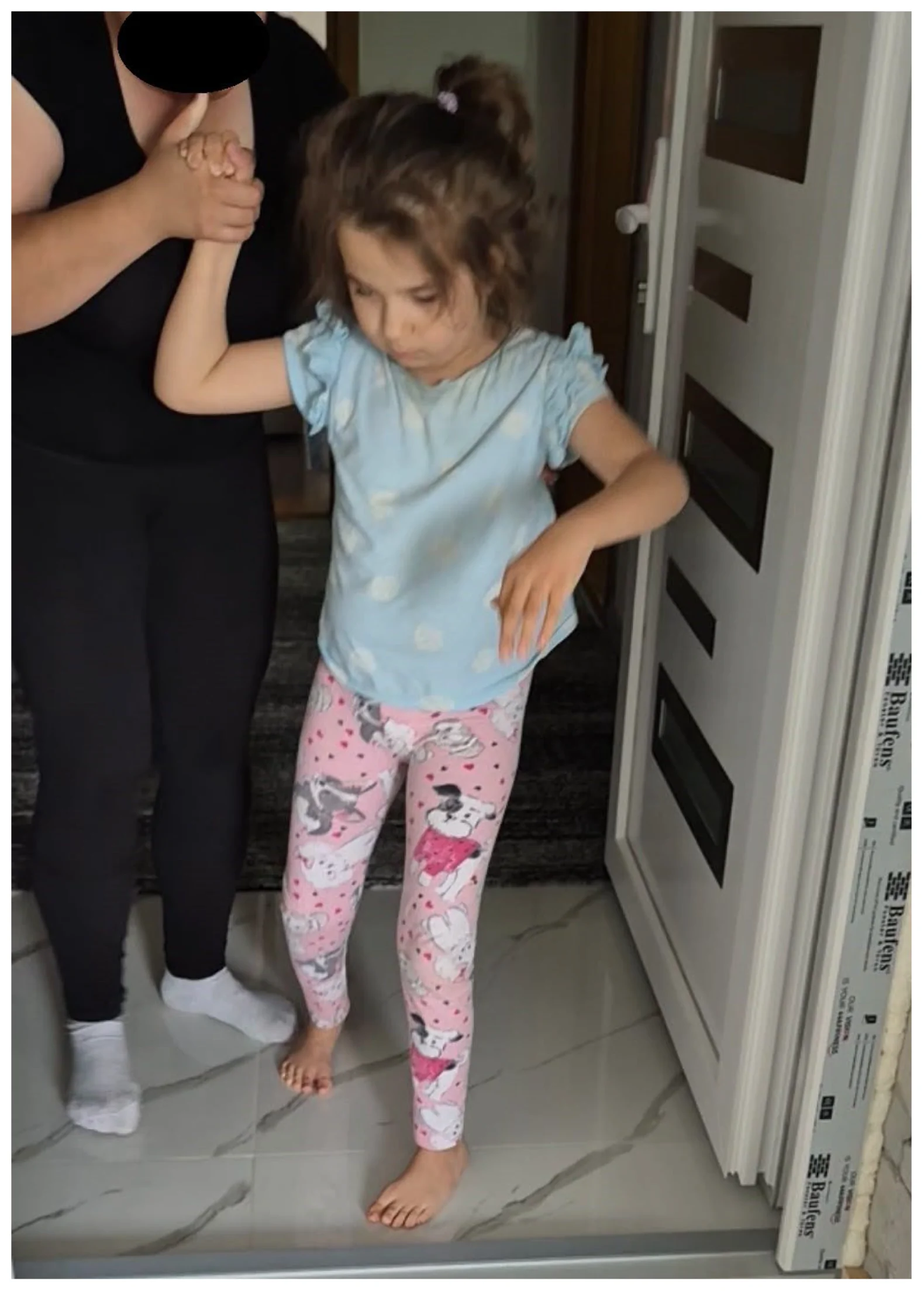
Ability to walk supported with one hand.

**Figure 10:**
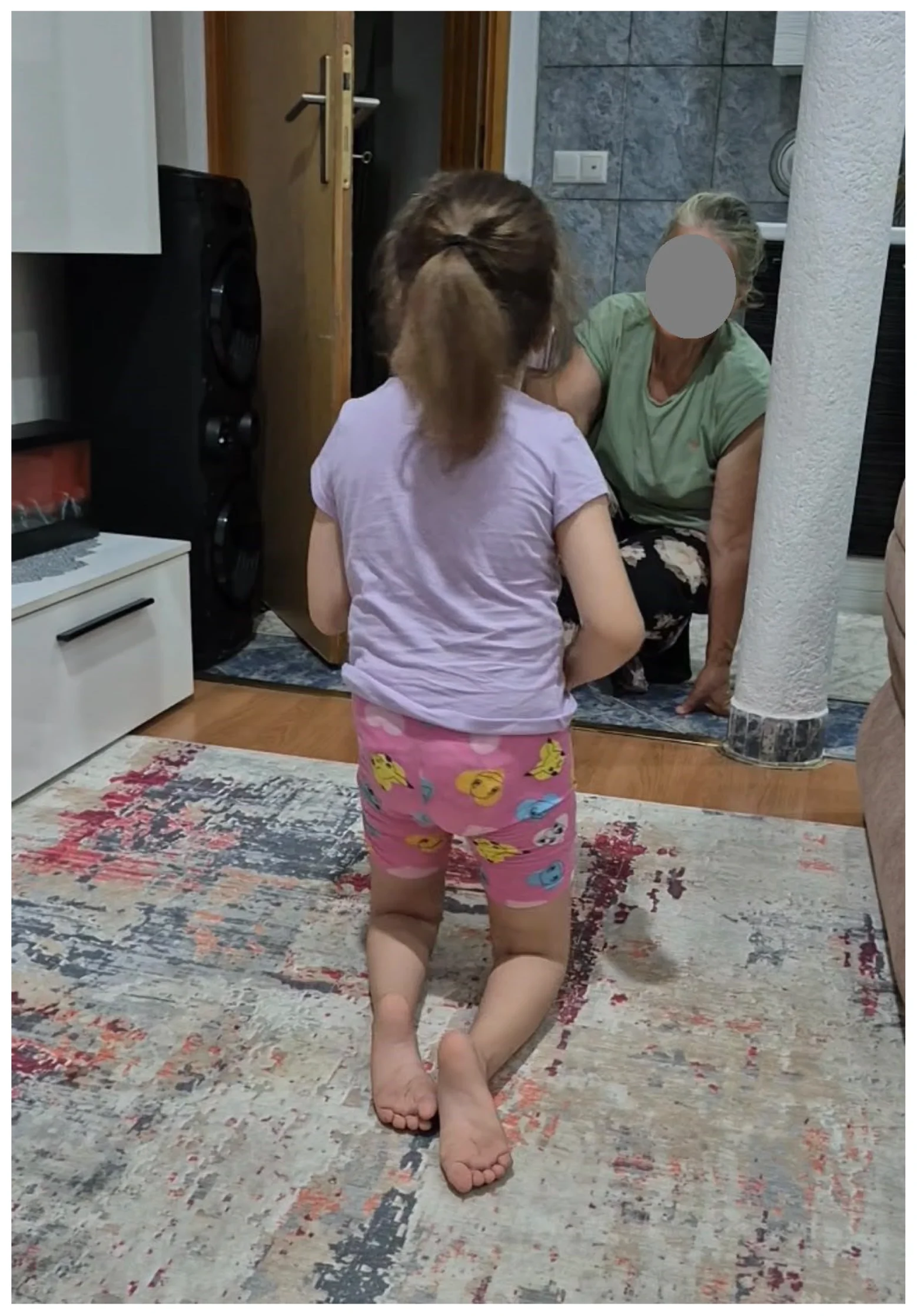
Ability to walk on knees.

Total and dimension percentage scores of GMFM-88 score are shown in Table 1. GMFM-66 interval scores are shown in Table 2, and graphically in Figure 1, Figure 2, Figure 3 and Figure 4, each showing results of one test. Figure 5, Figure 6, Figure 7, Figure 8, Figure 9 and Figure 10 show the ability of the child at a certain test, where Figure 5 is related to the first test, Figure 6 to the second, Figure 7 and Figure 8 to the third and Figure 9 and Figure 10 to the fourth.

The overall course of rehabilitation, including the therapeutic focus, the Vojta schedule, and the four assessment points, is summarized in Table 3.

**Table 3:** Overview of the rehabilitation course and assessment points from 15 months to 6 years 6 months of age. GMFM-88 total scores are shown at the four assessment points.

Age	Phase/Focus	Vojta	GMFM–88	Key Motor Change
1 year 3 months	Start; focus on gross motor function	Yes	10.8%	Prone position without reaching, rotating into prone position without leg dissociation
1 year 3 months ~3 years	Intensive motor phase	Yes	-	Commando crawling, pivoting, sideline grip
~3 years	Vojta discontinued; focus broadened to cognitive and communication domains	Stopped	-	Crawling on all fours
3 years 9 months	Second assessment	No	48.9%	Crawling on all fours, sitting
4 years 9 months	Third assessment	No	64.7%	Standing for 3 s, half-kneel to stand with support, trying side-stepping
6 years 6 months	Fourth (final) assessment	No	73.9%	Sideways walking, walking on knees, walking while holding hands up to 10 steps

## 5. Discussion

Framed within the ICF, the EIR-SPM engages the body-functions level (postural control, muscle tone, movement quality) but its intended and measured targets lie at the activity level: the GMFM-88 quantifies activities such as sitting, crawling, standing, and stepping. In this child, each newly acquired activity was accompanied by gains at the participation level—greater exploration, attention, and communication in everyday family life—so the program was not confined to impairment-level goals. EIR-SPM is built on the premise that intervention should be intensive and begin as soon as abnormal motor development is recognized, ideally within the first months of life, when experience-dependent plasticity is greatest [19]. Within the EIR-SPM framework, rehabilitation beginning before 3 months of age is regarded as very early, that beginning between 3 and 9 months as early, and that beginning after 9 months as delayed; in high-risk infants, the first 6 weeks of corrected age are considered the ideal time for initiation. Rehabilitation in this girl began at 15 months of age, which within the EIR-SPM framework is considered a delayed start [20]. At that point, she had generalized hypotonia with superimposed fluctuating episodes of hypertonia, poor postural control, and no independent sitting. Despite these less favorable starting conditions, the motor gains described in the results were observed over the course of therapy, although a single case cannot establish that they resulted from it. However, these observations are compatible with previously reported EIR-SPM findings in children at risk of developing CP [10] and in children with CP [16].

All developmental domains were addressed from the start, but the focus changed over time as the child’s needs changed. In the early phase, rehabilitation focused mainly on motor function, since this was where the child’s impairment was most severe; as independent locomotion through crawling was achieved and the child’s functional possibilities widened, increasing attention was directed toward cognitive stimulation, communication, and adaptive skills. Within this shift, Vojta therapy was discontinued after two years. Although the child continued to show good motor responses to the Vojta therapy, the rehabilitation team judged that the overall therapeutic load had become excessive and that the child was showing signs of fatigue, and this occurred at the time when the balance of the program was, in any case, moving toward the non-motor domains. Therapeutic components were retained, adapted, or withdrawn according to the child’s tolerance and current developmental priorities rather than a fixed protocol. Reducing the overall load at this stage also lessened the demands on the family, whose sustained involvement the program depended on.

Among children enrolled in the EIR-SPM, a distinction can be drawn between those in whom the motor domain is the dominant problem—such as high-risk infants or children with cerebral palsy without substantial impairment in other developmental domains—and children with global delay. In the former group, it is common practice to sustain intensive rehabilitation at a given developmental level for a prolonged period, with the aim of rendering the child’s spontaneous motor activity normal or as close to normal as possible. In a child with global delay, however, this logic must be weighed against the needs of the remaining developmental domains.

In the present case, the rehabilitation team accepted forms of locomotion that were at times not in an ideal pattern, while consistently directing attention to the quality of movement rather than to task achievement alone. This reflects a deliberate position on how motor learning operates in this population. In typically developing children, variability and self-generated trial-and-error exploration drive the selection of increasingly efficient movement. In children with atypical, fluctuating muscle tone and a constrained motor repertoire, however, this mechanism cannot be assumed to operate in quite the same way, or with the same efficiency: within the neuronal group selection framework, the reduced primary repertoire limits experiential selection, so that repeated trial-and-error may tend to reinforce the same atypical solution, as we have argued previously [16]. Left unaddressed, such stereotyped compensatory patterns may become entrenched and, over time, contribute to secondary musculoskeletal and functional consequences, as described in children with motor disorders [21]. We therefore regard active guidance of movement quality, within the child’s own spontaneous activity and play, as valuable in this context. Accordingly, certain milestones were encouraged to reach a sufficient quality of movement to support progression toward subsequent ones, and guidance was directed at discouraging stereotyped patterns that narrow the repertoire and impede progression-for example, persistent four-point crawling without adequate dissociation of the extremities (so-called bunny hopping), whose symmetry and rigidity limit the transition toward reciprocal crawling and upright locomotion. Each newly acquired form of independent locomotion was consistently followed by gains in the child’s other developmental domains, in exploration, attention, and communication rather than in motor function alone.

Within the EIR-SPM philosophy, prolonged and intensive rehabilitation of this kind is difficult to sustain unless it is family-centered, in the sense that it is delivered by the family in everyday life. This is consistent with the literature, in which family-centered care has been established as a best-practice model for child disability services internationally [22], premised on the belief that a child’s wellbeing and care needs are best supported within the family context through effective collaboration with professionals and services [23]. There is a growing international evidence base for the value of home-based service delivery and parent-mediated interventions in promoting the development of children with disabilities [24,25,26]. Moreover, interventions need to commence in the early years of the child’s life [27] and should not be dependent on the child receiving a formal diagnosis, as often happens due to resource constraints [28]. In the present case, rehabilitation was started at the first assessment at our clinic and continued without waiting for the diagnostic work-up to be completed.

A further feature of this case is the extent to which rehabilitation was distributed across the family. Beyond the parents, the child’s older sister and both grandparents were involved, each with a defined role agreed jointly between the family and the rehabilitation team. These roles ranged from motor facilitation to the use of everyday situations to stimulate attention, communication, and cognitive development. The deliberate involvement of siblings and extended family members has similarly been described as a component of a home-based early motor intervention program, intended to strengthen the family’s understanding of the child’s condition, support acceptance and family wellbeing, multiply opportunities for practice, and broaden the child’s everyday social contact [29]. In the present case, this distribution of roles allowed a high-intensity program to be sustained over more than five years without continuous institutional delivery. This flexibility was illustrated when the mother’s third pregnancy and the birth of a younger sibling temporarily limited her availability: the grandmother took over the main share of the home rehabilitation for that period, and the program continued without interruption. We use the term “family-centered” in the sense that the family delivered the rehabilitation and that rehabilitation goals were agreed jointly with them. We are aware that a fuller bio-psycho-social, family-centered model also encompasses the family’s own needs. In this case, the day-to-day therapeutic load was borne mainly by the parents, supported by the child’s older sister and grandparents and by monthly in-person reviews and online consultations between visits, which appeared to make this load sustainable. It may also carry practical implications: a program built on family education could reduce dependence on institutional care and, potentially, the associated economic burden, and may widen the range of settings in which rehabilitation can be sustained. In this case, therapy continued in the child’s home country between periodic visits abroad to the Polyclinic.

A factor that should not be overlooked is the motivation of the members of the rehabilitation team, among whom the parents are almost certainly the most highly motivated. Having a child with a disability places parents in the position of having to accept an unacceptable situation, and parental psychosocial difficulties may in turn affect the child’s rehabilitation process [30]. In our clinical experience, enabling parents and the wider family to participate actively in rehabilitation fosters a sense of shared purpose that may ease this process and contribute to better overall outcomes. Such involvement, however, must be weighed against the demands it places on the family. In families of children with global developmental delay, home activity programs have achieved high compliance—31 of 41 families were still using the program after seven months—with the degree of compliance depending on the support provided by the therapist and on family size [31]. The same study cautioned that parents maintaining high compliance appeared to be those most vulnerable to stress, and that therapists should remain aware of the considerable demands such programs place on mothers [31]. In the present case, the sharing of roles across several family members, combined with regular institutional follow-up and the availability of online consultation, may have mitigated this burden and contributed to the sustainability of the program.

This report is limited by its single-case design, the absence of a comparison group, and the still-incomplete etiological work-up, which precludes conclusions about the efficacy of the EIR-SPM. In addition, only the gross motor domain was followed with a standardized instrument; the gains described in the other domains rest on clinical observation, and the cross-domain effects suggested here should therefore be interpreted with caution. The GMFM-88 assessments were performed by a single examiner who was also involved in the child’s rehabilitation and was therefore not blinded; formal inter-rater reliability could not be evaluated, and the possibility of assessor expectation bias cannot be excluded. The use of a standardized, criterion-referenced instrument administered consistently by one experienced rater partly mitigates, but does not eliminate, this limitation. The family’s perspective and psychosocial context were not assessed with a formal instrument, and functional goals were not set collaboratively with the family; incorporating these would strengthen future evaluations of the EIR-SPM. A further consequence of the absence of a comparison group is that the change in GMFM scores across the period from 15 months to 6 years 6 months of age cannot be disentangled from expected developmental maturation; this is a logical rather than an empirical caveat, but it constrains any inference about the specific contribution of the intervention. Although no minimal clinically important difference (MCID) has been established for the GMFM in children with GDD, the magnitude of change can be compared with published thresholds for related populations. For the GMFM-88 total score, reported MCID values are 1.1–5.3% in acquired brain injury and 0.1–3.0% in cerebral palsy [32]. For the GMFM-66 total score, MCID thresholds in ambulatory children with CP range from 0.7 to 1.7 points for a medium effect and from 1.2 to 2.7 points for a large effect across GMFCS levels (0.8 and 1.3 points, respectively, for the overall sample) [33]. These MCID thresholds were derived in cerebral palsy and acquired brain injury and are therefore only an indirect reference for a child with GDD. More fundamentally, the GMFM is built around gross motor milestones that are largely acquired by about 5 years of age in typical development, so normal maturation contributes substantially to score change in early childhood [34]; the GMFM alone therefore cannot isolate the effect of the intervention from maturation, a limitation that applies with particular force to a single case followed from 15 months to 6 years 6 months of age. The increments recorded in this child (GMFM-88: +38.1, +15.8, and +9.2 percentage points; GMFM-66: +24.5, +6.1, and +5.1 points between successive assessments) exceed the upper bound of every published threshold at each interval. The clinical significance of the change is therefore not in question; what a single-case design cannot establish is the extent to which it reflects the intervention rather than developmental maturation. Set against this, the child’s undetermined etiology is itself relevant to the interpretation of outcome: in a prospective cohort of children with GDD reassessed at school age, a lack of an identified etiology predicted poorer motor outcomes [35]. That study used a different instrument (the Battelle Developmental Inventory) and reports group averages, not individual trajectories, so it is not a formal benchmark. Even so, the motor progress seen in this child is greater than the generally poor course described for GDD of undetermined etiology [35].

At baseline, measurable gross motor function was essentially confined to the lying and rolling and sitting dimensions of the GMFM-88, whereas at final assessment she scored across the crawling and kneeling, standing, and walking dimensions, having progressed from complete dependence on caregivers for every change in position to independent floor mobility, independent sitting in all positions, and independent standing with adequate postural alignment. Further reports and larger case series of children with GDD undergoing EIR-SPM are needed to establish whether comparable trajectories can be achieved in this population.

## 6. Conclusions

In this child with GDD of undetermined etiology, more than five years of intensive rehabilitation delivered according to the EIR-SPM were accompanied by substantial and sustained gains in gross motor function. Causality cannot be established in a single case, but the favorable course observed here is consistent with a potential benefit of the EIR-SPM in children with GDD that warrants further study, and suggests that meaningful progress remains achievable even when rehabilitation begins later than the period considered optimal within the EIR-SPM framework.

The magnitude of the observed change exceeded published thresholds of clinical importance for related populations and appeared more favorable than typically reported for GDD of undetermined etiology; a single-case design, however, cannot separate the contribution of the intervention from developmental maturation, so these comparisons should be interpreted as descriptive rather than confirmatory. Central to this was the family-centered structure of the program, in the sense that the therapy was delivered by the family: an intensive program of this duration would have been difficult to sustain through institutional delivery alone, and it was the parents, together with the child’s older sister and grandparents, who carried it into everyday life at home.

## Figures and Tables

**Table 1:** Results of Gross Motor Function Measure–88 (GMFM-88) testing.

GMFM Testing	Age of Child	GMFM-88 Score	Dimension A	Dimension B	Dimension C	Dimension D	Dimension E
1.	1 year 3 months	10.8%	49.0%	5.0%	0%	0%	0%
2.	3 years 9 months	48.9%	100.0%	80.0%	64.3%	0%	0%
3.	4 years 9 months	64.7%	100.0%	100.0%	83.3%	35.9%	4.2%
4.	6 years 6 months	73.9%	100.0%	100.0%	95.2%	59.0%	15.3%

**Table 2:** Recalculated Gross Motor Function Measure–66 (GMFM-66) interval scores.

GMFM Testing	Age of Child	GMFM-66 Score	Standard Error	Low 95% CI	Upper 95% CI
1.	1 year 3 months	19.7	2.2	15.3	24.1
2.	3 years 9 months	44.2	1.1	42.0	46.4
3.	4 years 9 months	50.3	1.2	47.9	52.7
4.	6 years 6 months	55.4	1.2	53.0	57.8

## Data Availability

Clinical findings used in this study may be made available from the corresponding author upon reasonable request and following approval by the institutional ethics committee. This would additionally require written parental consent and ethics committee approval.

## Abbreviations

The following abbreviations are used in this manuscript:

GDD	Global developmental delay
EIR-SPM	Early Intensive Stojčević-Polovina Rehabilitation Method
GMFM	Gross Motor Function Measure
GMAE-3	Gross Motor Ability Estimator
CP	Cerebral palsy
CI	Confidence Interval
MCID	Minimal clinically important difference
ICF	International Classification of Functioning, Disability and Health
